# Enhanced Apigenin Dissolution and Effectiveness Using Glycyrrhizin Spray-Dried Solid Dispersions Filled in 3D-Printed Tablets

**DOI:** 10.3390/biomedicines11123341

**Published:** 2023-12-18

**Authors:** Asma B. Omer, Farhat Fatima, Mohammed Muqtader Ahmed, Mohammed F. Aldawsari, Ahmed Alalaiwe, Md. Khalid Anwer, Abdul Aleem Mohammed

**Affiliations:** 1 Department of Health Sciences, College of Health and Rehabilitation Sciences, Princess Nourah bint Abdulrahman University, P.O. Box 84428, Riyadh 11671, Saudi Arabia; abalfaki@pnu.edu.sa; 2 Department of Pharmaceutics, College of Pharmacy, Prince Sattam bin Abdulaziz University, P.O. Box 173, Al-Kharj 11942, Saudi Arabia; mo.ahmed@psau.edu.sa (M.M.A.); moh.aldawsari@psau.edu.sa (M.F.A.);; 3 Department of Pharmaceutics, College of Pharmacy, Najran University, Najran 66433, Saudi Arabia

**Keywords:** 3D printing, apigenin, colon cancer, glycyrrhizin, spray-dried solid dispersions, printlets

## Abstract

This study aimed to prepare glycyrrhizin–apigenin spray-dried solid dispersions and develop PVA filament-based 3D printlets to enhance the dissolution and therapeutic effects of apigenin (APN); three formulations (APN1–APN3) were proportioned from 1:1 to 1:3. A physicochemical analysis was conducted, which revealed process yields of 80.5–91% and APN content within 98.0–102.0%. FTIR spectroscopy confirmed the structural preservation of APN, while Powder-XRD analysis and Differential Scanning Calorimetry indicated its transformation from a crystalline to an amorphous form. APN2 exhibited improved flow properties, a lower Angle of Repose, and Carr’s Index, enhancing compressibility, with the Hausner Ratio confirming favorable flow properties for pharmaceutical applications. In vitro dissolution studies demonstrated superior performance with APN2, releasing up to 94.65% of the drug and revealing controlled release mechanisms with a lower mean dissolution time of 71.80 min and a higher dissolution efficiency of 19.2% compared to the marketed APN formulation. This signified enhanced dissolution and improved therapeutic onset. APN2 exhibited enhanced antioxidant activity; superior cytotoxicity against colon cancer cells (HCT-116), with a lower IC50 than APN pure; and increased antimicrobial activity. A stability study confirmed the consistency of APN2 after 90 days, as per ICH, with an f2 value of 70.59 for both test and reference formulations, ensuring reliable pharmaceutical development. This research underscores the potential of glycyrrhizin–apigenin solid dispersions for pharmaceutical and therapeutic applications, particularly highlighting the superior physicochemical properties, dissolution behavior, biological activities, and stability of APN2, while the development of a 3D printlet shell offers promise for enhanced drug delivery and therapeutic outcomes in colon cancer treatment, displaying advanced formulation and processing techniques.

## 1. Introduction

Colon cancer is the second leading cause of morbidity and deaths globally, being the first leading cause among males and the second among females [1]. Colon carcinoma is a multiphase process that originates from a sequence of cellular and histopathological variations that involve different types of tumor suppressor genes and oncogenes. These genes lead to the transformation of normal and healthy epithelial cells in the colon into invasive carcinogenesis [2,3]. Various conventional strategies have been reported for the treatment of colon carcinoma, including chemotherapy, radiation therapy, and polypectomy and surgery [4]. However, these strategies (particularly chemotherapy) exhibit toxicity, as they not only target the cancerous cells but also affect the normal cells. These conventional strategies are usually strategically combined with targeted therapeutics, mostly for treating advanced stages of colon carcinogenesis [5]. 

In the last few decades, researchers have shown more interest and explored the anticancer potential of numerous plant-derived phytoconstituents or natural compounds, which have shown superior results in anticancer therapy. Amongst the several plant-derived phytoconstituents, flavonoids have gained immense interest due to their potential antioxidant and anticancer properties [6,7]. Flavonoids are secondary metabolites associated with the polyphenolic class. They are abundantly found in green tea, vegetables, fruits, and other plant sources and have shown significant chemotherapeutic actions with negligible toxicity [8]. It has been reported that proper intake of flavonoids significantly reduces the incidence of colorectal neoplasia; thus, flavonoids possess a chemo-preventive effect. A multi-centered case–control study described that appropriate intake of flavones, generally in high concentrations, diminished the occurrence of colorectal carcinogenesis [9,10]. 

**Figure 1:**
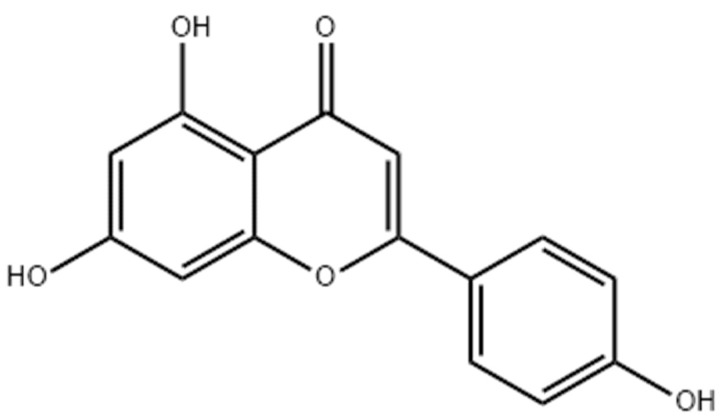
Chemical structure of apigenin.

Apigenin (APN), chemically known as trihydroxy flavone (3-OH group), is a naturally occurring solid flavonoid (Figure 1) with a crystalline nature. It is abundantly found in various natural sources, such as parsley, artichokes, chamomile, and spinach [11]. Various pharmacological activities of APNs have been reported in studies, including antioxidant, anticancer, anti-inflammatory, anti-diabetic, anti-microbial, and anti-stress properties [12]. APN has been explored more in the last few decades than other flavonoids due to its greater cytotoxicity and improved specificity against neoplastic cells than against normal and healthy cells [13]. Furthermore, studies have shown that the major anti-neoplastic mechanism related to APN is the apoptosis of neoplastic cells, as it inhibits the G2/M phase of the cell cycle in numerous neoplastic cells, as in colorectal cancer. APN majorly modulates some apoptotic proteins such as signal transducer and activator of transcription-3 (STAT3), phosphoinositide 3-kinase (PI3K), and extracellular signal-regulated kinase (ERK) [7,13,14,15]. Numerous in vitro and in vivo studies have stated the anticancer application of APN, for example, its capability to terminate cancerous cells by triggering cellular cycle arrest, stimulation of immune responses, cellular apoptosis, and autophagy [16]. APN has shown potential chemotherapeutic effects against various cancerous cells, including cells in the colon, skin, breast, thyroid, and prostate carcinoma, as well as cancerous cells in leukemia [15,17,18,19]. Although APN has imposing anticancer activity, its poor aqueous solubility and non-specific cell/tissue distribution remain challenges, deterring its application as a chemotherapeutic drug [20]. To date, various approaches have been established to overcome several limitations associated with APN delivery, especially polymeric nanoparticles, nanocrystals, and lipid-based nano-capsules [21,22]. Moreover, researchers have reported strategic novel nano-drug delivery systems that enhance its release, circulation time, cell uptake, and therapeutic effects [23]. 

Glycyrrhizin (GCN) is a glycoside derivative of glycyrrhetinic acid, also named glycyrrhizic acid; it is a saponin-like phytoconstituent mainly responsible for the sweet taste of *Glycyrrhiza glabra* (licorice). Licorice is widespread in Europe and is widely used, especially in South and North Africa. Furthermore, the Scandinavian region is one of the global leaders in the consumption of licorice candies. 

**Figure 2:**
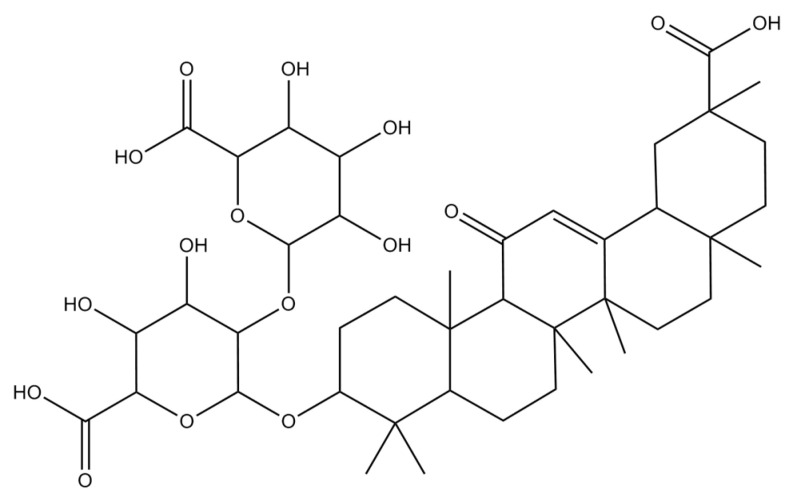
Chemical structure of glycyrrhizin.

GCN is an extensively used Chinese herbal medicine with potential therapeutic properties that include antioxidant, anti-tumor, anti-inflammatory, and antiviral properties [24]. GCN is a naturally occurring pentacyclic triterpenoid glycoside (Figure 2) isolated from root extracts of *Glycyrrhiza glabra.* It is also used as a major therapeutic agent in countries like Japan to effectively treat chronic viral hepatitis [25,26]. GCN induces apoptosis in various carcinogenic human cell lines, including cells in prostate cancer (DU-145 and LNCaP), hepatoma (HLE), leukemia (HL-60), stomach cancer (KATO III), and others [27,28,29].

Spray drying is a well-known engineering method that has been used in the design and formulation of amorphous solid dispersions, a potential approach to delivering poorly aqueous-soluble drugs [30]. Spray drying is an extensively used method for fabricating polymer/lipid-coated delivery systems for diverse purposes, including colon targeting [31]. The alteration of environmental situations, solution properties, or process parameters results in diverse formulation properties that enable the perfection of the functionality-associated properties of the finalized product. A few factors, such as the rate and amount of the drug and/or polymer solubility, regulate the rapid or extended onset of site absorption [30].

Phytotherapeutics play a crucial role in overcoming the limitations of conventional therapeutics in potential cancer therapy. However, these phytoconstituents have certain limitations as direct therapeutic agents due to poor aqueous solubility and permeability, which result in their low bioavailability [22]. Therefore, phytotherapeutics must be delivered using advanced drug delivery systems to overcome the limitations associated with these herbal drugs. 

Three-dimensional printing (3DP) has revolutionized the pharmaceutical sector and changed the way dosage forms are made; 3DP was first created for industrial use, but it has quickly matured into a promising technology. With the help of this breakthrough, 3D goods may be created on a digital design platform layer by layer. 

The FDA-approved Aprecia Pharmaceuticals’ “Spritam” was the first 3D-printed pill. The purpose of Spritam is to treat seizures brought on by epileptic diseases. Conventional oral dosage forms render adverse responses linked to drugs a “one-size-fits-all” strategy, particularly in elderly patients who have polypharmacy. A new study utilizing 3DP technology as an additive manufacturing technique indicates great promise for the production of individualized oral medicine prescriptions. Oral dosage forms may be produced quickly, consistently, and flexibly using 3DP techniques such as fused deposition modeling (FDM), selective laser sintering (SLS), and inkjet/binder jetting. The potential of pharmaceutical 3D printing to improve efficiency, accuracy, and personalization while cutting waste expenses is drawing interest [31]. In the present study, a robust formulation approach to developing glycyrrhizic-acid-coated apigenin-loaded solid dispersion through an ultrasonication-spray drying technique was reported. The optimized solid dispersions were evaluated for their synergistic and potential antioxidant, anticancer (colon carcinoma), and antimicrobial activity (*S. aureus*) through in vitro evaluation studies. Furthermore, the potential molecular mechanisms involved in the anti-colon cancer activity of the optimized formulation were evaluated.

## 2. Materials and Methods

### 2.1. Materials

The APN used was purchased from Beijing Mesuchem Technology Co. Pvt. Ltd. (Beijing, China), with a purity of ≥95.0%, while the glycyrrhizic acid (GCN) was procured from Azura Pharmaceuticals & Chemicals, Zaharababad, India. Ethanol and NaOH were purchased from Sigma-Aldrich, Darmstadt, Germany. All the other chemicals used were of analytical grade. Moreover, 3-(4,5-dimethyl-2-thiazolyl)-2,5-diphenyl-2H-tetrazolium bromide (MTT), Griess reagent, and 2′,7′-dichlorodihydrofluorescein diacetate (H2DCFDA; dichlorofluorescein diacetate) were procured from Sigma-Aldrich Co. (St. Louis, MO, USA). Mueller–Hinton agar (MHA), Nutrient Broth (NB), and Nutrient Agar (NA) media were the products of Difco, Fisher Scientific, New Hampshire, UK.

#### Development of Glycyrrhizin–Apigenin Spray-Dried Solid Dispersions

The development of glycyrrhizin–apigenin spray-dried solid dispersions (SDSDs) involved a meticulous and systematic process. Initially, three distinct batches of herbal glycyrrhizin–apigenin SDSDs—namely, APN1, APN2, and APN3—were prepared. This was achieved by sonicating and stirring a suspension composed of APN and GCN in different ratios of 1:1, 1:2, and 1:3, respectively. To initiate this process, APN (1 gm) and GCN (1–3 gm) with respect to the batch (APN1-3) were dissolved in a solvent mixture consisting of 60 mL of ethanol (C_2_H_5_OH) and 40 mL of 0.5% sodium hydroxide (NaOH). Subsequently, this suspension was dispersed with precision using a high-speed disperser (Model: 99224-2, Stanhope-Seta, Surrey, UK) for 2 h.

The resulting suspension was then employed for the spray-drying process within a controlled environment. This process was conducted in a hot air chamber to yield the three aforementioned batches (APN1, APN2, and APN3). The spray drying was executed using specialized equipment: a spray drying instrument (Model B-290 by Buchi Labortechnik, Flawil, made in Switzerland). The entire procedure was conducted under control parameters: a flow rate of 200 mL/h, an inlet temperature of 80 °C, an aspirator rate of 100%, and a solution feed rate of 20%. After the spray drying, the resultant products were carefully collected and preserved under vacuum conditions for further comprehensive characterization, biological evaluation, and rigorous stability studies [32].

### 2.2. Physicochemical Characterizations

#### 2.2.1. Process Yield

The yield of the spray-dried production was assessed by calculating the mass percentage of the output solids regarding the input solids. To determine the input solids, the composition weight of each batch (APN1–3) was utilized in calculations. A higher yield percentage indicates that a larger proportion of the input materials are being successfully transformed into the desired output solids. Conversely, a lower yield might suggest inefficiencies, losses, or suboptimal conditions during the production process.

#### 2.2.2. Apigenin Content Estimation

The determination of actual APN content in the SDSDs prepared using GCN was conducted using a Jasco UV500 spectrophotometer (Jasco spectrophotometer, Tokyo, Japan). SDSD (ANP1–3) sample amounts (5 mg) were weighed and dissolved in 10 mL of an aqua–ethanol solution. The absorbance of APN was measured in triplicate at a wavelength of 264 nm using a quartz cuvette. By applying linear regression analysis to the unknown samples, a comparison with the calibration curve APN content in each batch was performed. A calibration curve for APN was constructed using a stock solution by adding a known amount of ethanol and making standard solutions with concentrations ranging from 1 to 12 µg/mL. The calibration curve was plotted using the data absorbance (nm) at λ_max_ (264 nm) against concentration (µg/mL). The high coefficient of determination (R^2^ = 0.9976) indicates a strong correlation between concentration and absorbance [21].

#### 2.2.3. FTIR Spectroscopy

Pure APN, GCN, physical mixture (APN-PM), and three SDSD batches (APN1, APN2, and APN3) were triturated with KBr and pelleted with handheld compression. The pellet film was then placed in the holder and passed through the FTIR spectrum before the sample test instrument was aligned with the blank (KBr). All the spectrums were collaged and interpreted using the software of Jasco 6000 series FTIR spectrophotometry, Tokyo, made in Japan [30].

#### 2.2.4. Differential Scanning Calorimetry

All the samples under investigation (APN, GCN, APN-PM, and APN1–APN3) were crimped into the aluminum pan individually and placed against the reference (blank) pan. The calorimeter (Scinco Thermal analyzer model N-650, Seoul, made in the Republic of Korea) was supplied with nitrogen with a flow of 20 mL/min, and thermal ranged between 50 °C and 400 °C. The change in heat flow between the sample and reference indicated a phase transition, including melting and crystallization [32].

#### 2.2.5. Powder-X-ray Diffraction Analysis

X-ray diffraction (XRD) studies helped to determine the physical behavior and size of the particles in the powdered samples. All the samples (pure APN, GCN, APN-PM, APN1, APN2, and APN3) were evaluated for their physical behavior, whether they were amorphous or crystalline, and the possible interface was estimated using a Siemen D5000 Diffractometer [30].

### 2.3. Powder Characterization

#### 2.3.1. Angle of Repose

The Angle of Repose, an essential parameter in pharmaceutical research, was determined for the three distinct batches of glycyrrhizin–apigenin spray-dried solid dispersion, denoted APN1, APN2, and APN3. This analysis was conducted using a funnel method. Initially, the powders were poured through a funnel, and measurements were taken for the height and radius of the resulting powder cone that was formed. These measurements were subsequently utilized in a mathematical equation to calculate the Angle of Repose.

The Angle of Repose value obtained through this process was then subjected to a comparative analysis against the reference standards outlined in the United States Pharmacopeia (USP). This comparison aimed to assess the flow characteristics of the prepared glycyrrhizin–apigenin SDSDs concerning the established pharmacopeial criteria.

The determination of the Angle of Repose is a crucial step in evaluating the physical properties of solid pharmaceutical formulations. It provides valuable insights into the flow behavior of these formulations, which is essential in optimizing their manufacturing processes and ensuring their suitability for various pharmaceutical applications.

The mathematical equation used to calculate the Angle of Repose (*θ*) based on the height (*h*) and radius (*r*) measurements of the powder cone is as follows:

(1) $$\theta =\tan^{-1}\left(\frac{h}{r}\right)$$

#### 2.3.2. Carr’s Index:

In this study, the evaluation of the flowability and compressibility characteristics of SDSDs containing APN and GCN was conducted through the measurement of Carr’s Index.

The determination of Carr’s Index involved two critical steps: tapped density measurement and bulk density measurement. The tapped density, denoted as $\rho_{t}$, was quantified using a specialized apparatus (Pharma Test, model PT-TD300, Hainburg, Germany) that allowed for the mechanical tapping of a calibrated measuring cylinder filled with the SDSD (APN1–APN3) samples. Tapping continued until no further reduction in volume was observed. The bulk density, denoted as $\rho_{b}$, was determined by measuring the volume of a known mass of loose SDSD powder placed in a graduated cylinder.

Carr’s Index, expressed as a percentage, was then calculated using the following formula:

(2) $$\left(\%\right)=\frac{\rho_{t}-\rho_{b}}{\rho_{t}}\times 100$$

The resulting Carr’s Index values for each of the three SDSD batches were recorded and subsequently analyzed. A higher Carr’s Index suggests potential challenges in terms of flow and compressibility, while a lower Carr’s Index indicates better flow properties.

#### 2.3.3. Hausner Ratio

In this study, we implemented a comprehensive procedure to determine the Hausner Ratio (HR) for the three separate batches of SDSDs identified as APN1, APN2, and APN3. The HR was determined using a specialized apparatus, and this involved measuring both the bulk density (*ρ_d_*) and the tapped density (*ρ_t_*) for each batch.

The HR is calculated using the following formula:

(3) $$HR=\frac{\rho_{b}}{\rho_{t}}\;\;\;\;\;\;\;\;\;\;$$

The resulting values provided insights into the flow properties and compressibility of the SDSD formulations. A higher HR suggests potential challenges in terms of flow and compressibility, while a lower ratio indicates desirable flow properties, which are vital for efficient pharmaceutical manufacturing processes.

### 2.4. Design and Fabrication of 3D Printlet Shell

A round, empty printlet shell was designed using the computer-aided software Autodesk Fusion 360. The geometry of the 3D printlet shell was designed in a round shape with a cap and body of 12 mm outer diameter and 6 mm width. The cap was designed to get fixed inside the body, making the printlet shell appear as a single unit. The printlet shell design was saved as an Stl (Standard Triangle Language) file, which describes the 3D geometry of the design. This Stl file was transferred to 3D printer software that converts it to a printer-readable G-code (geometric code for computer numerical control). The G-code for the designed 3D printlet shell was formed using the 3D printer slicer software Repetier-Host V2.2.4 (with Slic3r Slicer). This G-code comprised all the slicing details for the design and printing parameter settings for the printer.

**Figure 3:**
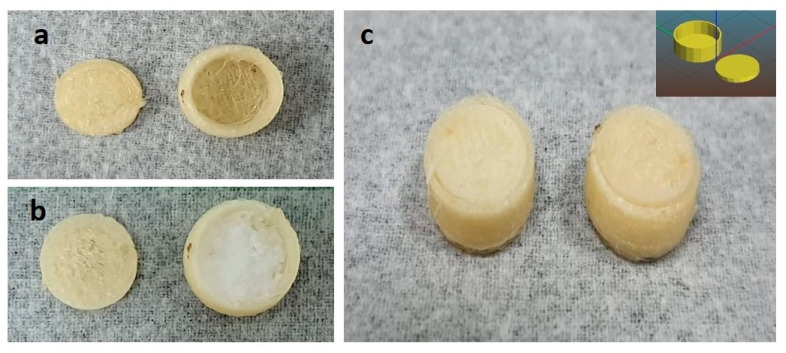
Demonstrates 3D printing process ((**a**) empty 3D printlet cap and body; (**b**) finished 3D printlet shell; and (**c**) after placing cap of the printlet shell).

The 3D-designed printlet was then printed with an FDM-based 3D printer (Easythred X1 Model). The printing settings for the layer thickness, shell thickness, and initial layer thickness were set to 0.2, 1.2, and 0.3 mm, respectively. The infill density was 15% with a rectilinear infill. The printing speed, travel speed, and bottom speed were set to 60, 100, and 20 mm/s, respectively. The low printing speed at the bottom facilitated the adhesion of the initial printed layers to the print bed. The formerly dried PVA filament was fed into the extruder of the 3D printer, wherein the filament was melted and extruded through the nozzle over a build plate, forming the designed 3D printlet shell in a layer-by-layer pattern. The diameter of the loaded PVA filament was 1.75 mm, while the extruder nozzle size was 0.4 mm. The nozzle temperature was set to 200 °C and the bed temperature to 60 °C. After the printing, the printlet shell body and cap were then removed from the printer bed, separated from the raft support, and subjected to filling and further characterization. The printing was carried out without retraction (Figure 3).

#### Dissolution Studies and Release Mechanism

The in vitro drug release studies of all the sample formulations (APN1, APN2, and APN3) and pure APN equivalent with 100 mg were filled into the fabricated 3D printlet. The sample-filled printlet was then placed into the dissolution medium containing phosphate-buffered solution (PBS; pH 7.4; 900 mL), maintained at 37 ± 3 °C, and paddled at (50 rpm) as per USP dissolution –II apparatus. At pre-determined time intervals, 5.0 mL of aliquots were withdrawn and substituted with freshly prepared PBS (pH 7.4). Further, the aliquots were assessed at 268 nm (λ_max_) through a UV-Vis Spectrophotometer (Jasco UV/Visible Spectrophotometer V-630, Japan). All the studies were performed in triplicate (n = 3). Additionally, the drug release kinetics and mechanism of the optimized formulation were estimated by screening the drug release data through various mathematical models, including zero and first order, Higuchi, and Korsmeyer–Peppas [32].

Moreover, mean dissolution time (MDT) and dissolution efficiency (DE) were also calculated. MDT is a critical metric in pharmaceutical research that indicates the rate of drug dissolution. It is calculated by dividing the area under the curve (AUC) of the cumulative percentage released (% CRs) versus the time curve by the total percentage of drug released (%D total) during the dissolution test. The formula for MDT is as follows:

(4) $$MDT=\frac{\sum_{j=1}^{n}t_{j}^{*}\;\Delta M_{j}}{\sum_{j=1}^{n}\Delta M_{j}}$$

This parameter facilitates the understanding of the dissolution behavior of drugs and formulations and enhances the optimization of drug delivery systems for enhanced therapeutic outcomes.

DE is a crucial parameter in pharmaceutical research that reflects the effectiveness of a drug’s release from a formulation. It is calculated by dividing the AUC by the maximum possible AUC. The formula for DE is as follows:

(5) $$D_{E}\left(\%\right)=\frac{\int_{t1}^{t2}ydt}{y100\;\left(t2-t1\right)}$$

where *j* is the sample number, (*n*) is the number of samples being dissolved, *y* is the percentage of drug dissolved and (*t*) is the time in minutes. The increased amount of medicine dissolved between *t* and *t*(*j* − 1) is (*M_j_*), and (*j*) is the halfway time between *t* and *t*(*j* − 1).

A high DE value indicates efficient and rapid drug release, which can lead to quicker therapeutic onset. In contrast, a lower DE may signify delayed or incomplete drug release, potentially affecting therapeutic efficacy. Therefore, DE serves as a valuable tool in formulating drug delivery systems that optimize drug release for improved patient outcomes.

### 2.5. Biological Evaluations

#### 2.5.1. Antioxidant Assay: DPPH Free Radical Scavenging Assay

The antioxidant activity of pure AG, optimized formulation, and ascorbic acid (standard) was examined using a DPPH free radical scavenging assay [33]. Initially, the DPPH stock solution (0.1 mM) was prepared by dissolving DPPH salt in the required amount of methanol, and then the solution was kept in a dark condition and at room temperature at 37 ± 2.0 °C. Then, all the test samples (50 μL) were individually treated with DPPH solution (100 μL) and kept in 96-well plates. Finally, the absorbance of the respective test samples was analyzed and estimated using a microplate spectrophotometer (λ = 517.0 nm) for 48 h, and the percent radical scavenging activity (%) results were plotted.

#### 2.5.2. Anticancer Study: Cell Viability Assay

The chemotherapeutic effects of pure APN and an optimized formulation were evaluated against the colon cancer cell line (HCT-116) using an in vitro MTT assay [34]. Initially, the colon cancer cells were passaged, incubated at 37 °C, and kept undisturbed overnight. On the day of the study, 100–120 μL of the previously cultured cell suspension with a cell density of ~5 × 10^4^ cells/mL was added to a 96-well plate. Each sample (pure APN suspension and optimized formulation APN2) with a volume equivalent (10 mg) was cautiously added into the respective wells. Subsequently, 100 μL of MTT solution (5% *w*/*v*) was added to individual treated wells and incubated for 24 h at 37 °C. Moreover, IC_50_ was also calculated using the MTT assay data, fixing it in the Quest Graph™ IC_50_ Calculator [34].

#### 2.5.3. Antimicrobial Assay

In the comprehensive antimicrobial assay, PureAPN (as the reference), Test-APN2, and Control-Betadine were utilized to assess antimicrobial activity across four distinct microorganism strains: *S. aureus*, *B. subtilis*, *E. coli*, and *C. albicans*.

The procedure commenced with the preparation of Petri dishes, each containing agar medium customized to meet the specific growth requirements of the intended microorganism strain. Subsequently, wells were created within the agar medium using a sterile cork borer. Following this, we evenly spread a standardized inoculum of each microorganism strain across the surface of the agar plates.

To assess the antimicrobial potential of Pure-APN, Test-APN2, and Control-Betadine, solutions were carefully introduced into their respective designated wells separately.

To encourage the growth of microorganisms and evaluate antimicrobial activity, the plates underwent incubation at temperatures and for durations tailored to match the characteristics of each microorganism strain. After the completion of the incubation period, the zone of inhibition surrounding each well was measured in millimeters (mm) using a calibrated ruler, and the results were subsequently plotted.

Subsequently, the zone of inhibition data underwent a one-way analysis of variance (ANOVA) to determine if statistically significant differences existed among the groups “Pure-APN”, “Test-APN2”, and “Control-Betadine”. The F-statistic and *p*-value were obtained from this analysis. To further pinpoint specific group differences, a Tukey’s Honestly Significant Difference (HSD) post hoc test was conducted. This post hoc test compared each pair of groups (Pure-APN vs. Test-APN2, Pure-APN vs. Control-Betadine, and Test-APN2 vs. Control-Betadine) in terms of their impact on the zone of inhibition, providing a comprehensive assessment of the statistical significance of these differences [35].

#### 2.5.4. Stability Studies

The optimized SDSD batch of 100 mg filled in a 3D printlet (APN2) was subjected to a stability test by being placed/stored at 40 °C/75% RH condition for 90 days [36]. After this, the dissolution test was performed, the cumulative release data from before and after stability studies were profiled, and the similarity index *f*_2_ value was calculated using the Moore and Flanner Equation (Equation (2)).

(6) $$f_{2}=50\times \;\log\left[\left\{1+\frac{1}{n}\sum_{r=1}^{n}wt\;\langle R_{t}-T_{t}\rangle \right\}\times 100\right],$$

where *n* = number of time-points, *R_t_* (references), and *T_t_* (test) is % drug released.

Moreover, the APN content percentage of the APN2 3D printlet was also calculated, as mentioned in the apigenin content estimation, in order to assess the possible degradation or interactions within the carriers and guide decisions on storage recommendations and shelf life [21].

## 3. Results

### 3.1. Physicochemical Characterizations

#### 3.1.1. Process Yield

The assessment of the process yield of this study, spray-drying production, was crucial in determining the efficiency of the transformation from input APN and GCN to output SDSDs. The yield amounts are weights for APN1 (1.61 gm), APN2 (2.73 gm), and APN3 (3.44 gm). This evaluation involved calculating the mass percentage of the output solids relative to the initial input solids. By utilizing the composition weight of each batch (APN1–3), we derived the input solids for these calculations. The calculated yield percentage served as a pivotal performance indicator for the spray drying process, reflecting its effectiveness [37].

Our results showed that the yields of the three batches fell within a notably high range, specifically ranging from 80.5% to 91%. More precisely, the process yielded 80.5% for APN1, 91% for APN2, and 86% for APN3. These figures highlight the remarkable efficiency of the spray-drying process, with a minimal proportion of the input materials being lost or wasted during production. Such consistently high yields indicate robust production conditions, and the set parameters can be utilized for mass production.

#### 3.1.2. Apigenin Content Estimation

The chemical assay results for APN content estimation in the three batches of SDSDs, APN1, APN2, and APN3, revealed that APN1 exhibited an APN content of 97.78%, while APN2 demonstrated a notably higher APN content of 99.78%. Meanwhile, APN3 displayed an APN content of 96.67%. These findings indicate the precise quantity of APNs present in each of the SDSD batches.

In the context of established pharmaceutical standards, specifically the USP monograph, the assay limit for any active substance content is set at 98.0–102.0%. The results obtained for APN1, APN2, and APN3 fell within this USP-defined acceptable range, indicating that the SDSD batches meet the stringent pharmacopoeia standards for APN content. This compliance reinforces the suitability of these batches for pharmaceutical use, as they align with the regulatory requirements and quality assurance criteria set forth using the USP method.

#### 3.1.3. FTIR Spectroscopy

**Figure 4:**
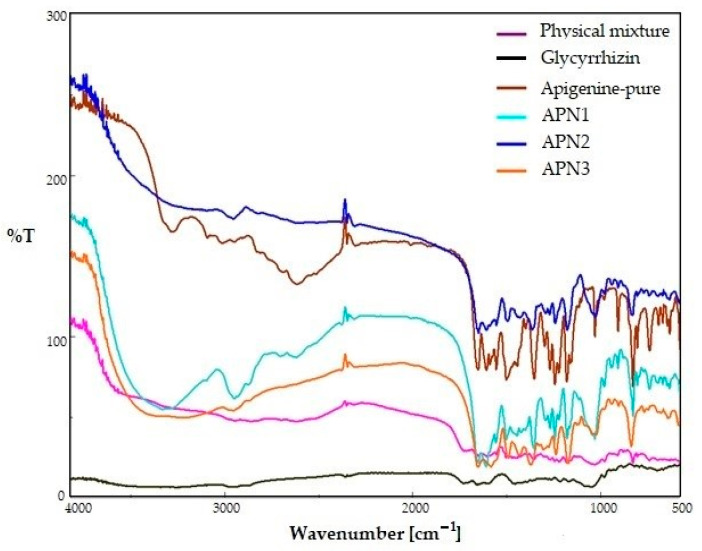
FTIR spectra of pure APN, GCN, SDSDs; APN1–APN3 and APN-PM.

The FTIR spectra of the pure APN and those of the three batches (APN1, APN2, and APN3), along with the physical mixture (APN-PM), were taken and collaged (Figure 4), followed by an interpretation of chemical interactions between APN and GCN. APN bands, a flavonoid compound of interest, reveal distinctive peaks that correspond to its specific functional groups and chemical bonds. The O-H stretch, which is indicative of the alcohol functional group (-OH), was characterized by a broad peak within the 3400–3200 cm^−1^ range. Meanwhile, the presence of a ketone functional group (C=O) was highlighted by a sharp peak at approximately 1725 cm^−1^. The aromatic nature of APN was unveiled through the C=C aromatic stretch, with the carbon–carbon double bonds (C=C) within its aromatic ring-producing peaks typically observed in the 1600–1450 cm^−1^ range. Additionally, the C-O stretch associated with ethers exhibited a peak in the vicinity of 1250–1050 cm^−1^. Lastly, the out-of-plane aromatic bending vibrations contributed to peaks within the 900–700 cm^−1^ range, elucidating the deformation of the aromatic ring. 

Interpreting the FTIR spectra of APN and SDSDs involves a detailed analysis of the specific peaks and absorption bands that correspond to the functional groups present in APN and GCN compounds along with the physical mixture (APN-PM). The FTIR spectrum of GCN has a peak at 3215.00 cm^−1^, which indicates O-H (hydroxyl) stretching vibrations. C=C (carbon–carbon double bond) stretching vibrations, typical in aromatic compounds, are suggested by the peak at 1597.57 cm^−1^., and a peak at 1036.28 cm^−1^ may be associated with C-O (carbon–oxygen) stretching vibrations. The region at 659.38 cm^−1^ corresponds to C-H (carbon–hydrogen) bending vibrations, a characteristic feature in alkanes. The FTIR spectrum of the physical mixture (APM-PM) of APN and GCN reveals distinct peaks at specific wavenumbers, providing valuable insights into its molecular composition. The prominent peak at 3260.60 cm^−1^ suggests the presence of O-H stretching vibrations, characteristic of alcohols and phenols. Peaks at 1434.42 cm^−1^ and 1031.61 cm^−1^ correspond to C-H bending vibrations in the aromatic ring and C-O stretching vibrations, respectively, suggesting the existence of ether or alcohol functional groups. Additionally, the peaks at 825.45 cm^−1^ and 738.02 cm^−1^ point to C-H out-of-plane bending and rocking vibrations in the aromatic structure. Peaks in the lower wavenumber range, such as 636.16 cm^−1^, 573.47 cm^−1^, and 497.47 cm^−1^, could be due to C-H bending vibrations in alkanes [38].

In the FTIR spectra of APN1, APN2, and APN3, for instance, several significant peaks could be identified and interpreted. The presence of a broad peak in APN1 at 3400–3200 cm^−1^ wavelength suggests the existence of alcohol functional groups (-OH), which correspond to the hydroxyl groups in both APN and GCN. Although such peaks diminished in APN2 and APN3, this reduction in the hydroxyl group intensity can be attributed to changes in the chemical environment within the SDSDs. Additionally, a sharp peak at approximately 1725 cm^−1^ indicated the presence of ketone functional groups (C=O) within the compounds. This peak was visible in all three batches of SDSDs and pure APN. Peaks in the range of 1600–1450 cm^−1^ are characteristic of aromatic compounds and confirmed the presence of aromatic rings in APN and GCN. Further analysis revealed peaks associated with the stretching vibrations of carbon–oxygen (C-O) bonds in ethers (1250–1050 cm^−1^), indicating the presence of ether functional groups. Lastly, peaks in the 900–700 cm^−1^ range are indicative of out-of-plane bending vibrations in aromatic rings, which provided additional confirmation of the aromatic structures within both compounds of all three batches. Given that all the major FTIR peaks characteristic of APN were present in the FTIR spectra of the three batches of SDSDs, it can be concluded that the preparation process of these batches did not significantly alter the fundamental chemical structure or the major functional groups of APN. The retention of these characteristic peaks suggests that APN remains chemically stable and that its essential properties are preserved within the SDSDs. This is a positive indication of the potential applications of these formulations, particularly in pharmaceutical or related fields where the integrity of the active ingredient is crucial for efficacy and safety.

#### 3.1.4. Differential Scanning Calorimetry

**Figure 5:**
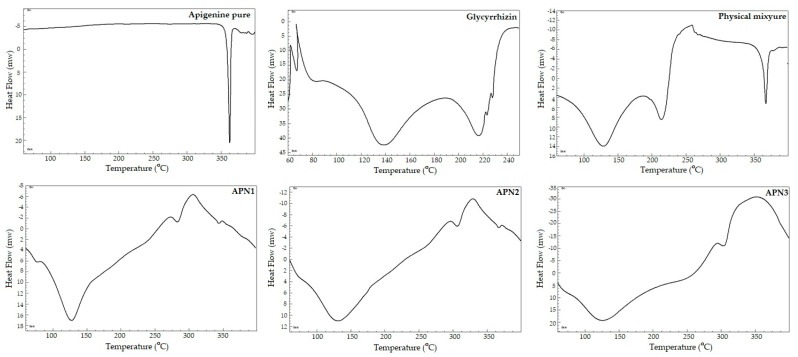
Thermograms of pure APN, GCN, APN-PM, and SDSDs APN1–APN3.

In the differential scanning calorimetry (DSC) thermographs of APN, a distinct sharp peak was observed at 362.5 °C, indicating its characteristic thermal behavior and crystalline nature. The DSC thermogram of pure glycyrrhizin revealed two prominent endothermic peaks that represent thermal events associated with the glycyrrhizin molecule. The initial endothermic peak observed at 135 °C suggests a thermal event, possibly corresponding to a phase transition that could be attributed to the transition from a solid to a liquid state. The subsequent endothermic peak observed at 210 °C indicates the chemical decomposition process. The DSC-APN-PM thermogram for the physical mixture exhibited two distinct endothermic peaks at 140 °C and 210 °C, which correspond to the thermal events associated with GCN. Additionally, a separate endothermic peak at 360 °C was observed, corresponding to pure APN. The results suggest that there is no molecular interaction between GCN and APN and no change in the solid state of materials with simple trituration (physical mixture). However, in the SDSD batches (APN1, APN2, and APN3), this sharp peak was notably absent, suggesting a change in the thermal behavior of APN during the spray-drying process. The absence of the APN peak in the SDSDs indicates that the APN is in an amorphous form within these formulations, as crystalline forms typically exhibit distinct thermal transitions. Instead, new thermal events were observed in the form of endothermic peaks at 125 °C and an exothermic peak at 320 °C within the SDSD batches. These thermal events can be attributed to the presence of GCN, which likely absorbed heat during these processes. Endothermic peaks, such as the one observed at 320 °C, are commonly associated with processes such as crystallization, chemical reactions, or decomposition. In this context, it suggests that GCN within the SDSDs underwent a thermal process at approximately 320 °C and released heat energy, possibly due to decomposition. Furthermore, the DSC analysis showed that the spray-drying process resulted in the transformation of APN from its crystalline form to an amorphous state within the SDSDs (Figure 5).

#### 3.1.5. Powder-XRD Analysis

The XRD spectrum of pure APN exhibited well-defined diffraction peaks at 2θ angles of approximately 20°, 30°, 35°, 50°, and 60°, characteristic of a crystalline material with a clear crystal structure. The X-ray diffraction (XRD) analysis of glycyrrhizin revealed a crystalline structure marked by a prominent and intense peak at 15° 2θ, indicating a well-defined crystallographic orientation or a significant structural feature within the material. The distinct sharpness of this peak implies that glycyrrhizin has a robust molecular arrangement. However, the presence of broader peaks at 20° and 35° 2θ suggests some degree of structural disorder or the presence of amorphous material. These wider peaks may indicate polymorphism or the existence of different crystalline forms within the GCN sample alone. The X-ray diffraction (XRD) peaks for APN-PM were observed at 12°, 14°, and 13°, along with the presence of an intense peak at 15°, in addition to peaks spanning the range of 20–30° in the physical mixture of APN and GCN, which collectively suggest a complex crystalline nature and reveal the intactness’ and crystal nature of both these components.

Moving on to the interpretation of the XRD patterns of the SDSD samples, APN1 and APN3 reflected prominent peaks at 2θ angles of approximately 28.5°, 47.5°, and 56.5°, as depicted in Figure 3. These peaks correspond precisely to the (111), (220), and (311) crystallographic planes of a face-centered cubic (FCC) crystal structure. Consequently, based on the positions of these diffraction peaks, it could be concluded that both APN1 and APN3 maintained an FCC lattice crystal structure similar to that of pure APN.

Conversely, when scrutinizing the XRD pattern of APN2, a significant reduction in the intensity of the peaks became evident. This reduction in peak intensity was indicative of the amorphous nature of SDSD APN2. Unlike APN1 and APN3, which retain a crystalline structure, APN2 displayed an amorphous structure. This distinction implies that APN2, with its amorphous nature, could represent an optimized formulation.

Amorphous formulations often exhibit enhanced dissolution rates and improved effectiveness due to their higher solubility compared to crystalline forms. Therefore, it is plausible to infer that APN2 may offer improved effectiveness and enhanced dissolution, making it a promising candidate for optimized drug delivery with significant implications for its potential applications in the drug development process, particularly concerning bioavailability and therapeutic efficacy.

#### 3.1.6. Powder Characterization

##### Angle of Repose

The Angle of Repose is a critical parameter used to evaluate the flow properties and cohesiveness of both coarse and fine powders. The Angle of Repose for the three distinct batches of APN-loaded SDSDs was calculated. APN1 exhibited an Angle of Repose measuring 48.67°, indicating relatively favorable flow properties and cohesiveness. Conversely, APN2 displayed a slightly lower Angle of Repose (46.28°), implying improved flow properties and reduced cohesiveness in comparison to APN1. In stark contrast, APN3 demonstrated a significantly higher Angle of Repose (56.31°), signaling relatively inferior flow properties and greater cohesiveness. These Angle of Repose values yield valuable insights into the flow characteristics of the SDSDs. For instance, APN2 stands out with the most favorable flow properties among the three batches, which can substantially contribute to enhanced manufacturability and the uniformity of dosage form production [39].

##### Carr’s Index

Carr’s Index values are dependable indicators of the flow properties of the investigated SDSDs. Higher Carr’s Index values are associated with poorer flow properties, indicating reduced compressibility and flowability, which can impede the filling of SDSDs in the 3D printlet and various manufacturing processes. Conversely, lower Carr’s Index values are desirable, as they suggest enhanced flow properties crucial for efficient and consistent production.

APN1 notably exhibited a Carr’s Index of 66, which was significantly higher than the Carr’s Index value observed for both APN2 and APN3 (52). This substantial difference underscores that APN1 possesses comparatively inferior flow properties and is less amenable to compression/filling in comparison with APN2 and APN3.

##### Hausner Ratio

The HR is a crucial parameter for assessing the flow properties of APN-loaded SDSDs. In this study, HR values were determined for the three different batches, namely APN1, APN2, and APN3. APN1 exhibited the most favorable HR (0.34), indicating excellent flow properties and compressibility. Conversely, both APN2 and APN3 displayed slightly higher HR values (0.48), signifying excellent flowability of SDSDs.

#### 3.1.7. Design and Fabrication of 3D Printlet Shell:

All three batches of SDSDs were filled into the 3D printlet, with 100 mg in each PVA shell. APN1 was composed of APN and GCN in a 1:1 ratio, containing 50 mg of APN and 50 mg of GCN. APN2 was composed of 1:2 ratios, i.e., 33.3 mg of APN and 66.6 mg of GCN, while APN3 in a 1:3 ratio contained 25 mg and 75 mg of APN and GCN, respectively [40].

#### 3.1.8. Dissolution Studies and Release Mechanism

**Figure 6:**
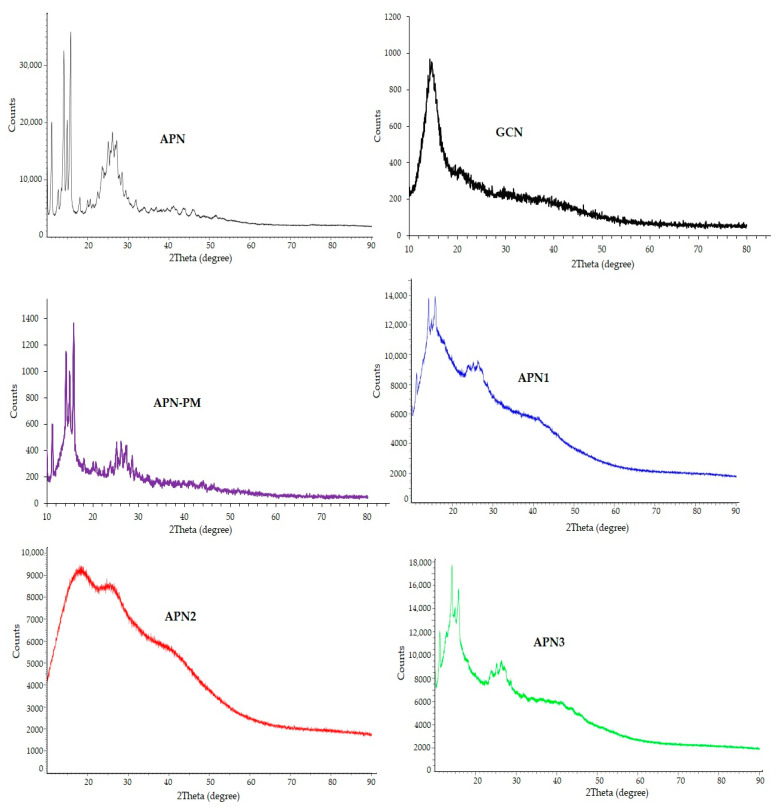
X-ray diffraction (XRD) patterns of pure APN, GCN, APN-PM, and SDSDs; APN1–APN3.

**Figure 7:**
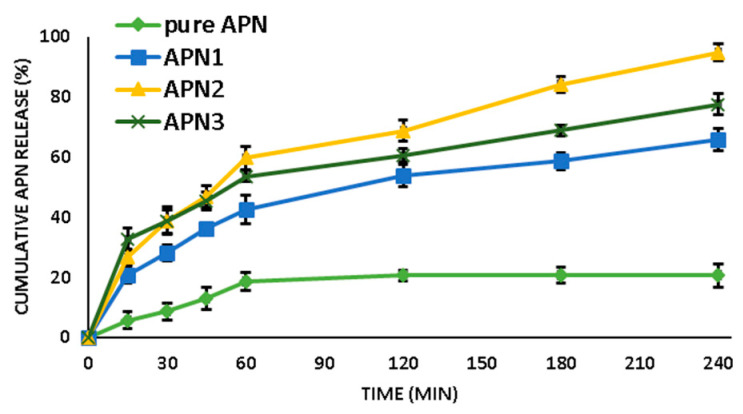
Cumulative APN release behavior of pure APN, APN1, APN2, and APN3.

As shown in Figure 6, APN2 exhibited superior release behavior compared to all other printlets, with a maximum drug release of 94.65 ± 1.34%; however, the release of pure APN was 20.68 ± 1.67%. Moreover, APN2 showed an initial burst release within the first 80 min, followed by continuous and improved release behavior for up to 240 min. It was noticed that the APN release behavior was significantly increased by micelle formation with GCN added to the SDSDs (Figure 7).

The dissolution study data provides valuable insights into the release profiles of different formulations. For instance, pure APN demonstrated limited release in the initial 15 min, with only 4.58 ± 2.8% released. Over time, there was a gradual increase in release, reaching approximately 22.68 ± 3.87% at 240 min. Altamimi’s investigation, as outlined in the study published in “Advanced Powder Technology”, elucidates the solubility properties of APN in water at 37 °C, revealing a solubility level of approximately 16 μg/mL.

In contrast, APN1, the SDSD 3D printlet with a 1:1 ratio of APN to GCN, exhibited a faster release compared to pure APN. At 15 min, 26.86 ± 2.67% of APN was released, and this continued to 67.63 ± 3.67%, significantly surpassing pure APN. This enhanced release can be attributed to the solubilizing effect of GCN, which enhances APN’s solubility in the dissolution medium. The presence of GCN in SDSD formulations appears to enhance solubility and subsequent release, with APN1 displaying the fastest release of complex APN.

APN2, which featured a 1:2 ratio of APN to GCN, exhibited an even more enhanced release profile. At 15 min, 28.32 ± 2.65% of APN was released, and it gradually increased over time, reaching an impressive 96.37 ± 2.87% at 240 min. The higher proportion of GCN relative to APN in this SDSD likely contributed to improved release kinetics due to complete micellization.

Several studies have demonstrated improved dissolution and bioavailability of drugs when combined with GCN, including sildenafil and curcumin. GCN has been shown to enhance drug absorption and penetration into cells. It can also increase drug absorption significantly in supramolecular complexes with GCN, which can facilitate the passive transport of drug molecules through cell membranes.

Similarly, APN3, with a 1:3 ratio of APN to GCN, demonstrated a release profile with 34.87 ± 3.67% of APN released at 15 min, reaching 79.83 ± 3.53% at 240 min. The relatively higher proportion of GCN in this formulation may also have contributed to the decreased rate of APN release compared to APN2.

It is worth noting that in APN1, the proportion of GCN may not have been sufficient to form micelles, thus resulting in reduced release compared to that in APN2 and APN3. At lower concentrations, GCN molecules exist as individual entities; however, in APN3, the higher GCN concentration led to increased micelle formation. A high concentration of GCN can potentially lead to the formation of excessive micelles or other aggregates that may hinder drug release.

Dissolution findings highlight the significant influence of GCN on APN release from SDSDs, with the potential for tailoring release profiles to optimize drug delivery systems. APN2 in the 3D printlet formulation holds promise for enhancing APN dissolution as well as for therapeutic outcomes.

The release kinetics data (represented by the calculated kinetic parameters ZO, FO, HC, KP, and n) provide insights into the release mechanisms of APN from different SDSDs. These parameters help us understand the behavior of APN release from the SDSDs that contain GCN.

The zero-order kinetics (ZO) parameter describes the release rate when the rate of drug release is constant over time. An R^2^ value close to 1 indicates that the release follows a zero-order kinetic model. In this study, the three formulations, APN1, APN2, and APN3, had R^2^ values of 0.806, 0.849, and 0.75, respectively. This suggests that APN release from these SDSDs was relatively constant over time, indicating that drug release is not dependent on the concentration of APN within the SDSDs.

First-order kinetics (FO) represents the release rate when the rate of drug release is proportional to the remaining drug concentration. In this study, the R^2^ values were 0.913, 0.968, and 0.917 for APN1, APN2, and APN3, respectively, indicating that APN release was influenced by the concentration of APN within the SDSDs, with higher concentrations resulting in faster release.

The Higuchi model (HC) parameter represents the square root of time vs. drug release, indicating a release mechanism where drug release is controlled by diffusion through a matrix or carrier material. In this study, the R^2^ values were 0.971, 0.983, and 0.942 for APN1, APN2, and APN3, respectively. This suggests that APN release follows a Higuchi model, where diffusion through the SDSD matrix plays a significant role in controlling drug release.

The Korsmeyer–Peppas Model (KP) parameter describes the release mechanism when drug release follows a power–law relationship with time. “n” is another parameter in the Korsmeyer–Peppas model, and it provides information about the release mechanism.

In APN1, the R^2^ value of 0.98 suggests a relatively high release rate, i.e., APN1 released its drug content relatively rapidly. The “n” value of 0.41 indicates a non-Fickian diffusion mechanism, suggesting that the drug release was not purely driven by diffusion but that it also involved other factors, such as swelling or erosion of the dosage form.

Similar to APN1, APN2 also has a high R^2^ value of 0.983, which fits the Higuchi model, indicating a sustained and relatively fast release rate. The “n” value of 0.44 also suggests non-Fickian diffusion, again indicating that factors other than pure diffusion contribute to drug release. These results showed that formulation followed rapid drug diffusion in the presence of water molecules, followed by stable diffusion of the encapsulated drug for a prolonged time.

APN3 had a slightly higher R^2^ value, indicating a fast release rate. However, the “n” value of 0.31 suggests a more pronounced non-Fickian diffusion mechanism. This shows that non-diffusion factors played a significant role in the drug release from APN3.

The release exponent, “n”, characterizes the mechanism of drug release. For Fickian diffusion, “n” is close to 0.5, while values between 0.5 and 1.0 indicate non-Fickian diffusion. Studies on release kinetics suggest that APN release follows non-Fickian diffusion, indicating a combination of diffusion and other release mechanisms.

In summary, the release kinetics data indicate that APN release from the SDSDs predominantly followed a combination of first-order kinetics, the Higuchi model, and non-Fickian diffusion. This suggests that the release of APN is influenced by both its concentration within the SDSD matrix and diffusion through the matrix, with the presence of GCN enhancing the release kinetics [31,38]. This comparative release profile indicates that APN2 had the most efficient and substantial release of APN compared to the other formulations tested, including APN1 and APN3.

**Figure 8:**
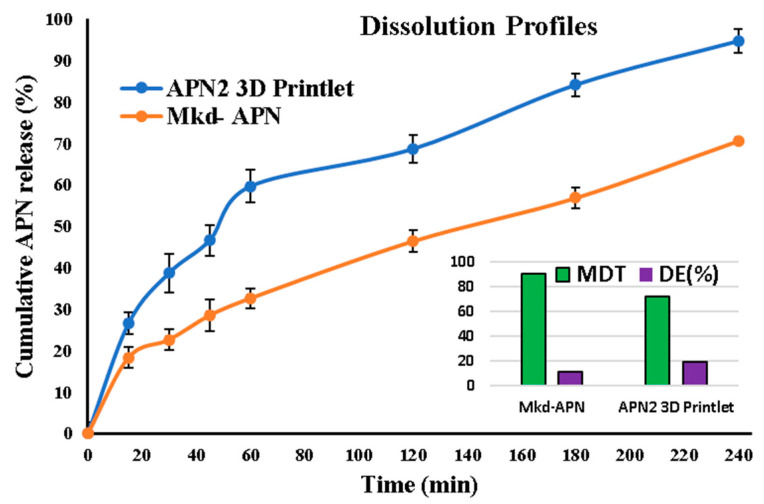
Dissolution profiles of APN2 (3D printlet) and Mkd-APN (marketed APN) with MDT and dissolution efficiency.

The dissolution profiles of APN2 (3D printlet) and marketed APN (Mkd-APN) over various time intervals are presented in the dissolution profiles, along with their respective MDT and DE (%) values in fir (Figure 8).

The dissolution profiles of APN2 (3D printlet) and Mkd-APN were compared at various time points to assess their release behavior. Notably, APN2 exhibited a significantly higher and faster rate of dissolution compared to Mkd-APN, as reflected in the lower MDT value of 71.80 min for APN2 compared to 90.17 min for Mkd-APN. This shows that APN2 reaches its maximum dissolution more rapidly than Mkd-APN.

Furthermore, the DE (%) values confirm the superior dissolution characteristics of the APN2 3D printlet. APN2 SDSDs filled in the 3D printlet demonstrated a DE of 19.2%, signifying that a higher proportion of the APN was efficiently released within the given time frame compared to Mkd-APN, which had a DE of 11.6%. This substantial difference in DE underscores the enhanced release behavior of the APN2 3D printlet, which can potentially lead to quicker therapeutic onset and improved patient outcomes.

The dissolution profiles, MDT, and DE values collectively indicate that APN2 (3D printlet) exhibits superior dissolution properties compared to Mkd-APN. These findings hold promise for optimizing drug delivery systems as well as enhancing therapeutic outcomes. Therefore, they warrant further investigation and potential clinical applications [40,41].

#### 3.1.9. Biological Evaluation Studies

##### DPPH Radical Scavenging Activity Assay

**Figure 9:**
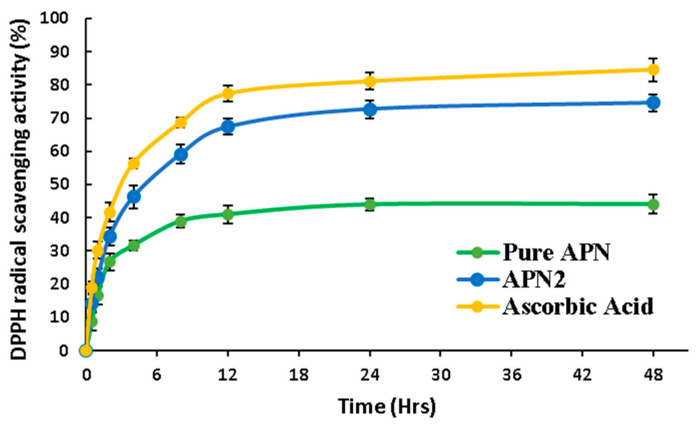
DPPH assay of pure APN, optimized formulation (APN2), and ascorbic acid (standard) for 48 h.

The antioxidant potential of pure APN and an optimized formulation (APN2) was determined using a DPPH assay (Figure 9). The DPPH radical scavenging assay is a simple, swift, cost-effective, and extensively used approach for evaluating antioxidant activity. Although the DPPH assay includes the transmission of hydrogen atoms, the primary chemical reaction is actually an electron transfer reaction [39]. In our study, the optimized formulation (APN2) showed potential antioxidant activity with similar results to ascorbic acid (standard). Ascorbic acid, which served as the standard antioxidant, displays the highest initial DPPH radical scavenging activity, which steadily increased, reaching 84.56 ± 3.52% after 48 h. Pure APN and APN2 exhibited a maximum DPPH radical scavenging activity (%) of 44.07 ± 2.82 and 74.56 ± 2.65, respectively. Hence, there was approximately a 1.69-fold increase in antioxidant activity for APN2 compared to pure APN. All the samples exhibited concentration-dependent activity [39].

##### Anticancer Study: Cell Viability Assay

**Figure 10:**
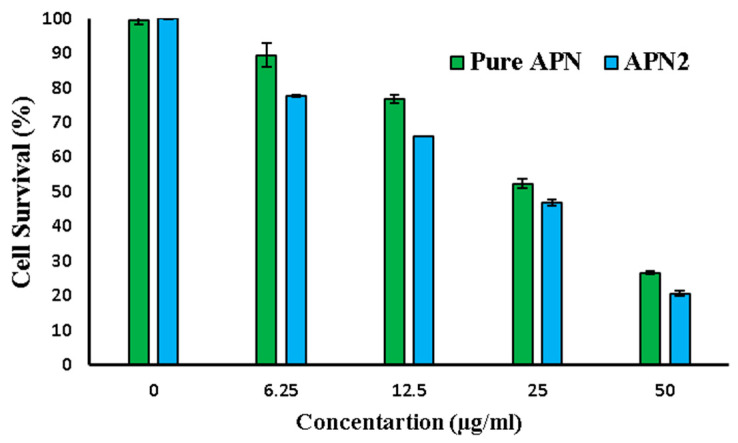
Cell survival percentage and concentration of pure APN and APN2.

The results of the cell viability assay offer valuable insights into the potential anticancer effects of both APN and the optimized APN2 SDSD formulations. This assay assessed the cell survival percentages across various concentrations of pure APN and optimized APN2 SDSDs, with results summarized in Figure 10. It becomes evident that both APN and APN2 SDSDs exhibit a concentration-dependent anticancer effect on cell survival. As concentrations increase, cell survival generally decreases, indicating their capacity to effectively inhibit cancer cell growth. Notably, when comparing APN to APN2 and considering standard deviations (SD), APN2 displayed lower average cell survival, suggesting a potentially more consistent anticancer effect across different experimental conditions.

Furthermore, we obtained IC_50_ values as well as fold-change data for both APN and APN2 SDSDs. IC_50_ values provide a quantitative measure of the concentration required to inhibit cell survival by 50%, with lower values indicating greater potency. Our data showed that APN2 had a lower IC_50_ value of 14.60 µg/mL, while APN had a slightly higher IC_50_ value of 18.37 µg/mL. This indicates that APN2 exhibits a more robust response to inhibiting cancer cell survival compared to APN.

The fold-change data, which quantifies the relative difference in potency between APN2 and APN, shows an approximately 1.26-fold increase in the potency of APN2 compared to APN. This substantial enhancement in the anticancer potential of APN2 aligns with the trend observed in the IC_50_ values, where APN2 demonstrates greater potency. Furthermore, the lower IC_50_ value and higher potency of APN2 suggest that it may require lower concentrations for effective cancer cell inhibition, potentially reducing side effects and enhancing the safety profile of the formulation.

In the assessment of the correlation between concentration and cell survival, both pure APN and optimized APN2 SDSD formulations display strong negative correlations. Pure APN exhibits a Pearson’s Correlation Coefficient (R) of −0.988, while optimized APN2 SDSDs show an R of −0.9723. Importantly, however, both formulations achieve statistical significance with *p*-values well below the 0.05 threshold, underscoring highly significant correlations between concentration and cell viability. The calculated *p*-values for pure APN and optimized APN2 SDSDs (0.001575 and 0.005601, respectively) further reinforce the substantial relationship between concentration and cell survival for both formulations. These findings collectively support the potential of both APN and APN2 SDSDs as effective anticancer agents, with APN2 demonstrating enhanced potency and lower IC_50_ values, making it a promising candidate for further therapeutic development.

Both APN and optimized APN2 SDSDs show concentration-dependent anticancer effects, with APN2 displaying enhanced potency and lower IC_50_ values, making it a promising candidate for further development in cancer therapy. The strong negative correlations and statistical significance in concentration–cell survival relationships support their potential as effective anticancer agents [14,42].

##### Antimicrobial Assay

**Figure 11:**
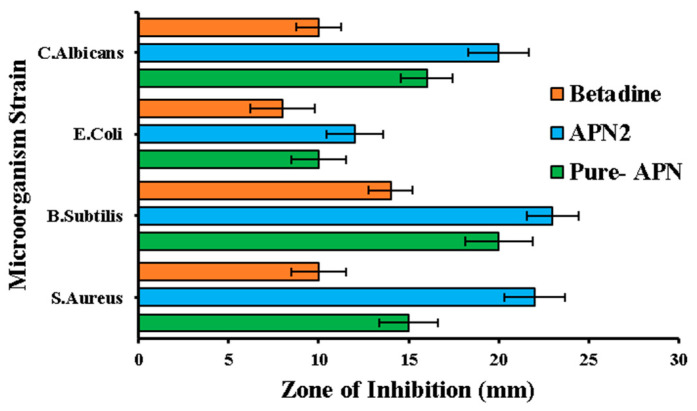
Zone of inhibition studies of pure APN and optimized formulation (APN2).

In the assessment of antimicrobial activity, Pure-APN (reference), Test-APN2, and Control-Betadine were examined against four different microorganism strains (*S. aureus, B. subtilis*, *E. coli*, and *C. albicans*). The zone of inhibition (measured in millimeters) provided insights into their effectiveness, with SD utilized to assess within-group variability (Figure 11).

APN2 demonstrated superior performance against *S. aureus*, exhibiting a substantial zone of inhibition (22 mm; SD: 1.65) that significantly surpassed those of Pure-APN (15 mm; SD: 1.68) and Betadine (10 mm; SD: 1.54). Similarly, for *B. subtilis*, APN2 displayed enhanced antimicrobial activity, with a significant 23 mm inhibition zone (23 mm; SD: 1.87), while Pure-APN achieved 20 mm (SD: 1.43) and Betadine 14 mm (SD: 1.23) inhibition zones. In the case of *E. coli*, susceptibility varied, with APN producing a 10 mm zone of inhibition (SD: 1.56) that was slightly overshadowed by that of APN2 (12 mm; SD: 1.54), while Betadine yielded an 8 mm zone (SD: 1.78). Lastly, for *C. albicans*, APN2 displayed a robust zone of inhibition measuring 20 mm (SD: 1.45), notably exceeding that of Pure-APN (16 mm; SD: 1.67), while Betadine matched the measurement for Pure-APN with a 10 mm zone (SD: 1.23).

To ascertain statistical significance, a Tukey’s HSD post hoc test was conducted following a one-way ANOVA. The ANOVA results revealed a statistically significant variance in the zone of inhibition among the Pure-APN, APN2, and Betadine groups, with an F-statistic of 16.83 and a *p*-value of 0.002. Subsequently, Tukey’s HSD post hoc test illuminated significant distinctions between the groups: Pure-APN vs. APN2, Pure-APN vs. Betadine, and APN2 vs. Betadine. Each of these pairings exhibited a noteworthy difference (*p* < 0.05) in their impact on the zone of inhibition. These findings underscore that all three groups possess distinct antimicrobial properties with statistically significant variations in their efficacy concerning the zone of inhibition.

The antimicrobial study highlights the varying antimicrobial effectiveness of Pure-APN, APN2, and Betadine against the tested microorganism strains. APN2 emerges as a potent antimicrobial agent in certain scenarios, with the statistical analysis confirming its significance. Therefore, APN2 SDSDs filled in a 3D printlet can be an effective antimicrobial besides having other therapeutic effectiveness [40,41,42,43].

#### 3.1.10. Stability Studies

**Figure 12:**
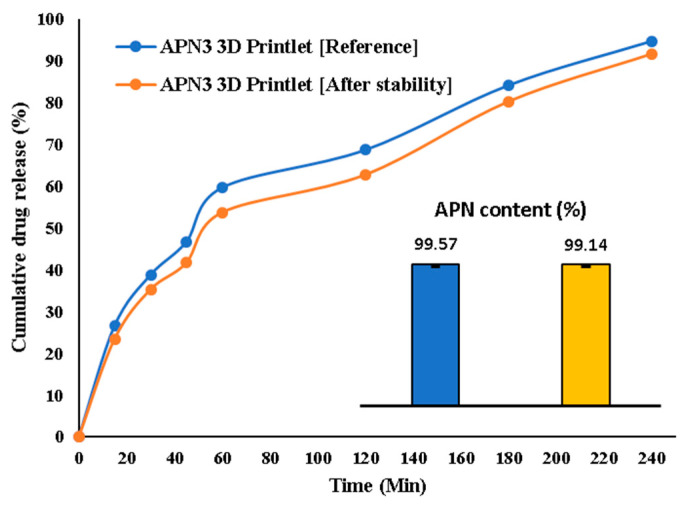
Stability studies of optimized 3D printlet (APN2) at accelerated thermal condition.

Similarity index results of the dissolution profiles of a 3D printlet (APN2) before and after undergoing stability testing (Figure 12). The results indicate an *f*_2_ value of approximately 70.59, which is a measure of the similarity between the two dissolution profiles [44,45].

As reported by Muqtader et al., an *f*_2_ value of 100 indicates identical dissolution profiles, and an *f*_2_ value of 50 or more is generally considered a good similarity, as suggested by Moore and Flanner [44].

In this stability test, the calculated *f*_2_ value was 70.59. This value is significantly above the threshold of 50, indicating a high level of similarity between the dissolution profiles before and after the stability test. This means that despite the storage conditions at 40 °C/75% RH for 90 days, the drug release behavior of the formulation (APN2) 3D printlets did not undergo substantial changes and remained stable under the specified storage conditions. The analysis of apigenin content in the APN3 3D printlet before and after stability studies revealed that APN content before stability studies was (99.57 ± 0.29%), while after stability studies, the mean content was (99.14 ± 0.24%). The calculated paired *t*-test resulted in a *t*-value of approximately 1.98 and a *p*-value of 0.104, indicating that the observed difference is not statistically significant at a conventional significance level of 0.05. These findings suggest that the APN3 3D printlet formulation effectively maintains APN content under the tested storage conditions (Figure 12). Such stability is crucial in pharmaceutical development because it ensures the consistent and predictable performance of the drug product over time.

## 4. Conclusions

In summary, this research centered on formulating spray-dried solid dispersions (SDSDs) of apigenin (APN) and glycyrrhizic acid (GCN), followed by the development of PVA-based 3D printlets and comprehensively characterizing their physicochemical attributes, dissolution kinetics, biological functionalities, and stability. The findings highlighted the favorable features of these SDSDs, particularly APN2, including satisfactory yield, flowability, assay (%), and the compatibility of APN with GCN, as evidenced via FTIR, DSC, and XRD analyses. Moreover, the formulations exhibited enhanced dissolution profiles, improved biological activities, and robust stability, positioning them as promising candidates for pharmaceutical and therapeutic applications. The developed APN2 3D printlet emerges as a potentially effective dosage form for the efficient delivery of apigenin (APN) to achieve its therapeutic effects.

## Data Availability

Data are contained within the article.
